# Serious bicycle crash injury in chiropractic practice – a case report of delayed diagnosis

**DOI:** 10.1186/s12998-016-0121-z

**Published:** 2016-11-01

**Authors:** Lars Uhrenholt

**Affiliations:** 1 Department of Forensic Medicine, Aarhus University, Palle Juul-Jensens Boulevard 99, 8200 Aarhus N, Denmark; 2 Nortvig & Uhrenholt Kiropraktisk Klinik, Jens Baggesens Vej 88A, 8200 Aarhus N, Denmark

**Keywords:** Bicycle traffic crash, Cervical spine fracture, Delayed diagnosis, Chiropractic practice

## Abstract

**Background:**

Bicyclists are vulnerable road users and are at risk of serious spinal injury if involved in traffic crashes. In Denmark approximately 25 bicyclists are killed each year and some 20.000 bicycle related casualties are registered in the National Patient Registry each year. In addition to these figures, a large number of casualties remain unregistered despite injury. Many of the casualties will consult chiropractors in primary practice with or without preceding evaluation in the established emergency care facilities. Therefore, chiropractors are expected to be able to proficiently evaluate these patients clinically and radiologically in order to ensure the best possible patient care.

**Case presentation:**

This report involves a middle-aged female who consulted several physicians following a collision with a motor vehicle while riding a bike. Despite clinical symptoms and consequent examinations she suffered from inadequate diagnostic evaluation until a radiological examination was performed 18 days following the injurious crash identifying unstable cervical spine fractures.

**Conclusions:**

The presented case is an example of the serious spinal injuries bicyclists may suffer when involved in high-energy traffic crashes despite wearing a bicycle helmet. The case report highlights the need for relevant clinical (including radiological) decision strategies when dealing with trauma patients in chiropractic practice. This involves the direct access to radiological procedures with no unnecessary delay when indicated as in most trauma cases. Furthermore, clearly defined and easy accessible referral schemes from primary care settings to emergency departments must be available to the chiropractic physician. Chiropractors are clinically competent to examine and diagnose, including radiologically evaluate, patients who have been injured in traffic crashes. Hence, chiropractors may contribute to the diagnosis, management and rehabilitation of spinal injured patients following many types of crashes and accident, including bicycle crashes.

## Background

In Denmark, biking is extremely popular for recreational purposes, sports and commuting. However, bicyclists can be regarded as vulnerable road users and consequently at risk of personal injury when involved in a road traffic crash. These crashes may be “solo” crashes or involve other parties, e.g. cars, bicycles or pedestrians. The mechanism of trauma significantly influences the types of lesions sustained by the casualty. In the perspective of injury prevention measures the bicyclist has limited options. The most common protective device is a bicycle helmet which has a well-documented effect on preventing serious head injuries [1–5]. However, there is limited scientific information concerning the effect of wearing a bicycle helmet and the risk of neck injuries [1–3, 6].

According to the National Patient Registry (Landspatientregisteret) approximately 20.000 Danes are injured on bicycles each year causing them to consult an emergency departments (ED) [7]. In comparison, only 10 % of these are registered in Denmark Statistics which is a national database based solely on police records when addressing traffic crashes [8]. Hence, casualties who do not contact the emergency department or become registered by the police should be added to the reported numbers. Therefore, the number of injured bicyclists is probably much higher than the official numbers, and this is most likely a worldwide phenomenon [9]. All fatalities are recorded which amounts to approximately 25 bicyclist each year equivalent to 15 % of all road traffic fatalities in Denmark [10, 11]. The Danish Traffic Safety Commission for 2013–2020 has defined bicycling as a focus area in order to improve our understanding of this underreported area of traffic injury and safety [9, 12, 13].

When injured, the bicyclist sustains injuries of a wide range of severities. For the majority of severely injured casualties immediate contact to the emergency department will take place. Rarely, these patients will contact a chiropractic physician directly. In some cases, presumably very few, a clinical evaluation at the emergency department will have been insufficient whereby clinically important conditions remain undisclosed. Some of these patients may consult a chiropractor in a primary care facility. Less severely affected individuals are more likely to contact a chiropractor directly.

This case report presents a middle-aged female who consulted several physicians following a bicycle crash before a radiological evaluation at a primary care chiropractic facility revealed serious cervical spine fractures. The purpose of this paper is to remind the clinicians of the challenges that primary care may face when consulted by newly injured bicyclist complaining of a variety of symptom severities.

## Case presentation

**Fig. 1:**
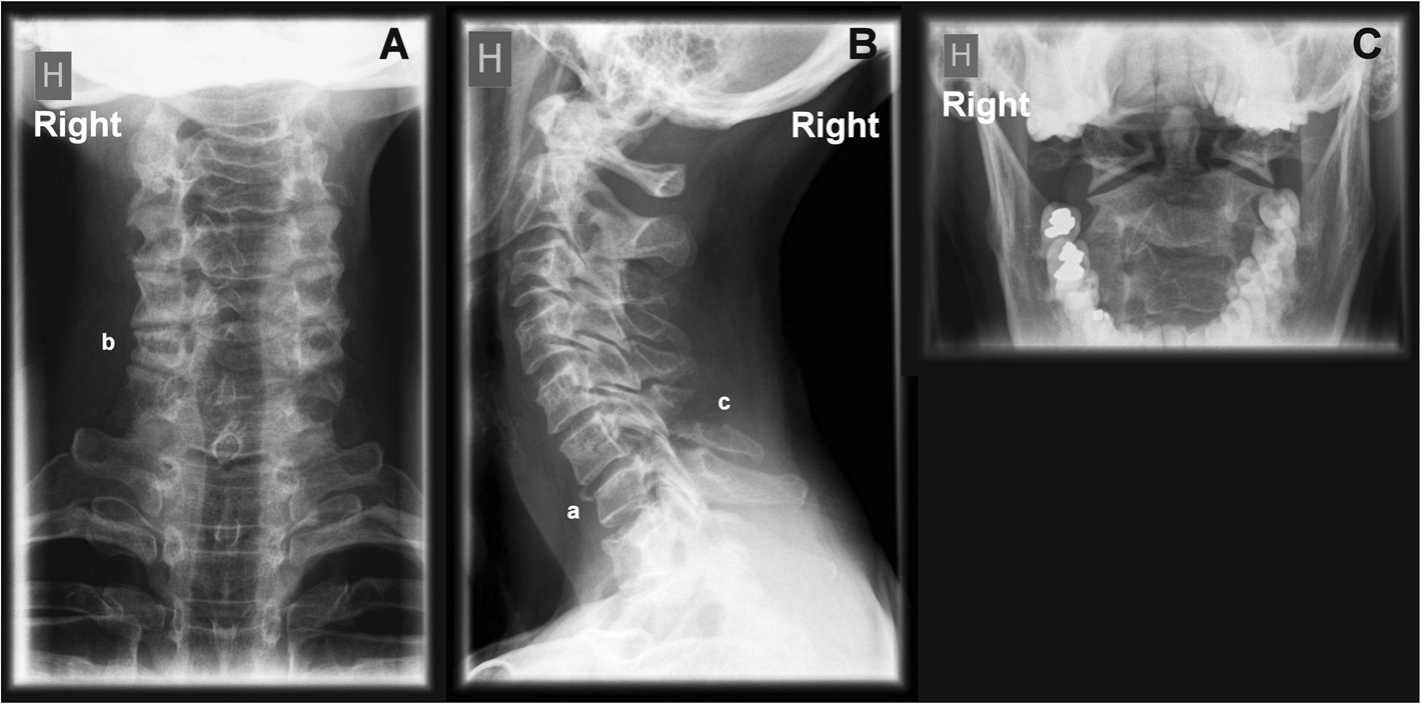
First diagnostic images (x-rays) of the cervical spine. Figure 1 shows a cervical spine series consisting of AP lower cervical (APLC) (**A**), lateral cervical (**B**) and AP open mouth (APOM) (**C**). These X-rays were the first diagnostic images of the cervical spine of the 49 year old bicyclist, taken at the chiropractor’s office 18 days following her traffic crash. The x-rays reveal an acute kyphotic angle between C6-C7 and a minor spondylolisthesis of C6 (3–4 mm) with suspicion of a fracture dislocation of the C6-C7 facet joints. There is a fracture of the anterior part of the vertebral body of C7 (a). There is reduced height of the articular column on the right side at C6 with suspicion of a fracture (b). There is a splitting fracture of the spinous process of C6 (c)

**Fig. 2:**
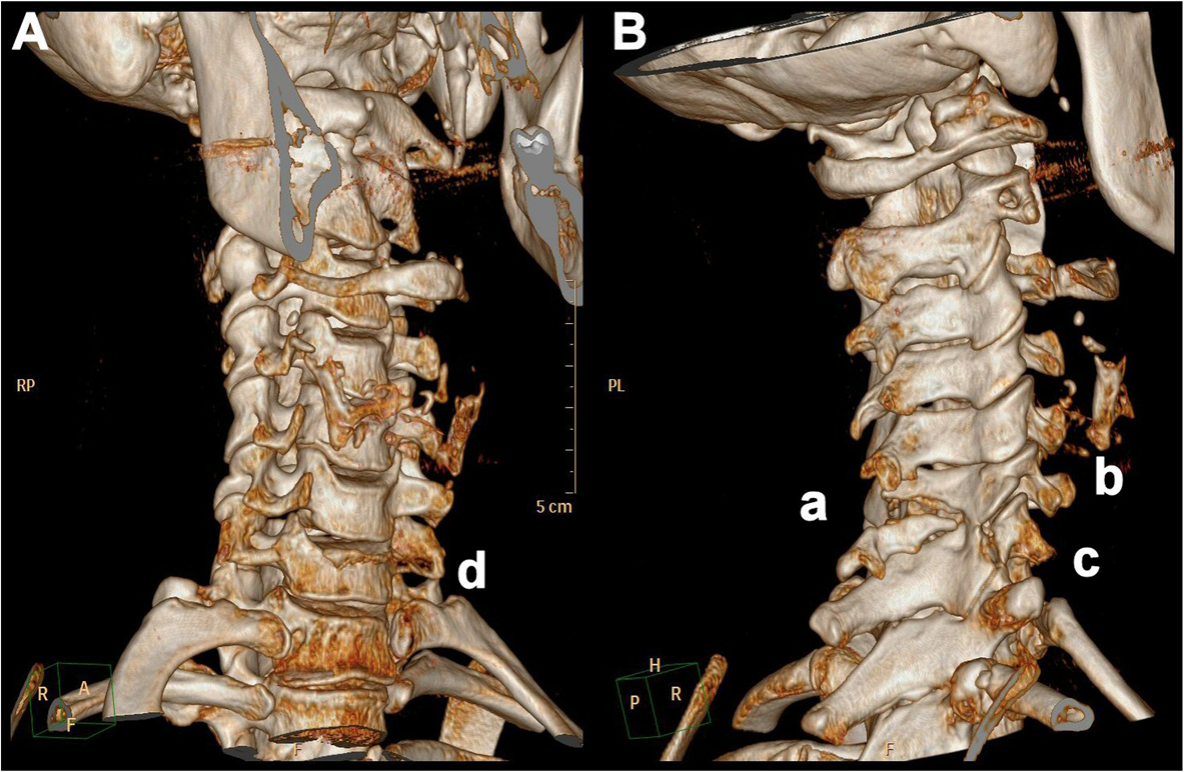
First Computed Tomography images of the cervical spine. Figure 2 shows the first Computed Tomography scanning obtained on the same day as the conventional x-rays in Fig. 1. The two images are 3D reconstructions of the original CT images with a slice thickness of 1 mm, where **A** is viewed from an anterior right angle and **B** is viewed from a posterior right angle. There is clear evidence of a fracture of the spinous process of C6 (a). The height of the articular column on the right side at C6 is reduced due to a fracture affecting the articular column (b). There is a fracture of the transverse process of C7 on the right (c). The vertebral body height of C7 is reduced at the anterior aspect (d). Please note that the figure does not illustrate all identified injuries

**Fig. 3:**
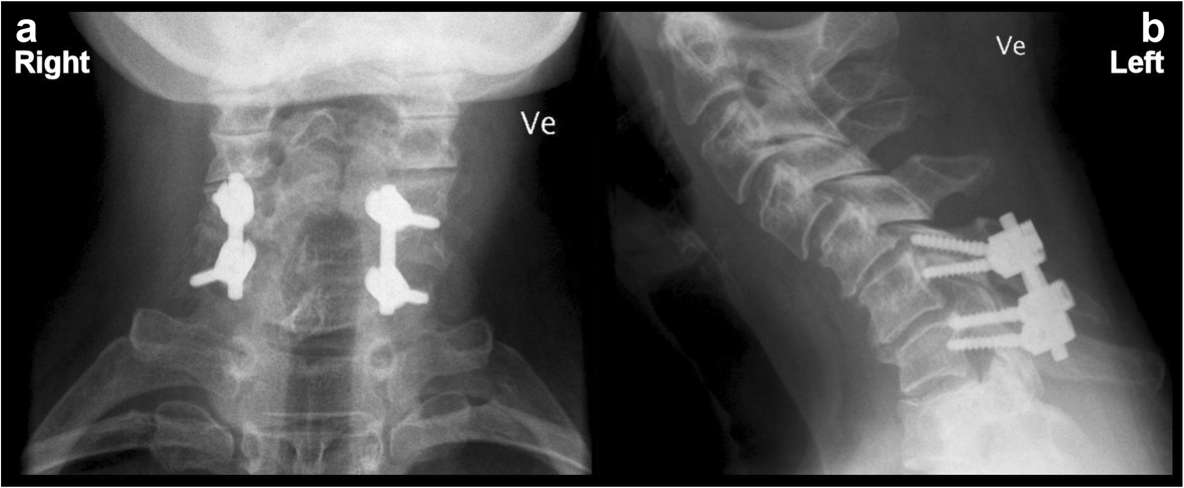
Post surgery control x-rays of the cervical spine. These two x-ray images ((APLC) (**a**) and lateral cervical (**b**)) were obtained at the hospital approximately 3 months following stabilizing osteosynthesis of the cervical spine. The images show a kyphotic angulation in the lower cervical spine with well positioned pedicle screws including longitudinal bars at both sides joining C6 to C7. There are no signs of osteolysis or loosening at the surroundings of the screws and the fractures appears to have healed accordingly

A previously healthy 49 years old female was biking wearing a bicycle helmet and not under the influence of any medication, drugs or alcohol. She was going at moderate speed (20–25 kph) as she entered an intersection. In the opposite direction a passenger car was preparing for a left hand turn and while doing so the car collided with the oncoming bicyclist. The bicyclist tumbled over the bonnet and windshield of the vehicle and landed on the ground on her left shoulder. There were no additional collisions. She recalled hitting her head against the ground and she did not lose consciousness. The inside padding of the helmet was broken after the crash. She could stand immediately afterwards although she suffered from acute neck pain, pain in the left shoulder and pain in the left knee. She observed bleeding from the left knee and bruises/abrasions on the legs but not the hands, arms or face. The police attended the scene and an ambulance was requested. However, the casualty decided to go home after the crash. After 3 h she nonetheless visited the Emergency Department (ED). At the ED a medical examination revealed a minor laceration injury to the left medial knee which was treated accordingly. The cervical spine was examined clinically without diagnostic imaging and no treatment was initiated. After a few days the patient visited the General Practitioner (GP) who referred her to a chiropractor. During the first consultation at the chiropractor’s office a few days later, she was examined clinically without diagnostic imaging and she received treatment of the cervical spine including manipulative therapy, which provoked the neck pain. The patient consequently terminated further treatment. The following week, 18 days after the initial traffic crash, she consulted another chiropractor. She now complained of increasing stiffness in the neck, frequent neck pain (not constant), and pain in the right scapula and upper arm. There were minimal symptoms from the left knee. Due to the history a cervical spine x-ray series (APLC, APOM and lateral) was performed initially at the chiropractor’s office (Fig. 1). This revealed an acute kyphotic angle between C6-C7 and a minor spondylolisthesis of C6 (3–4 mm) with suspicion of a fracture dislocation of the C6-C7 facet joints. There was a fracture of the vertebral body of C7. There was reduced height of the articular column on the right side at C6 with suspicion of a fracture. There was a fracture of the spinous process of C6. Due to the findings on the initial radiographs no additional x-rays were taken and no clinical examination was performed. Instead, the chiropractor immediately contacted the patient’s GP on the phone for the purpose of an acute referral to the ED which the GP enacted. A letter was mailed to the GP with the radiological interpretation and a CD-ROM containing a copy of the x-rays was given to the patient. The patient received no treatment at the chiropractor’s office. Later the same day a Computed Tomography (CT) scanning was performed at the Neuroradiological Department at the local University Hospital (Fig. 2), confirming the x-ray description with additional findings including a fracture of the superior endplate of Th1, a fracture through the transverse process of C7 on the right and an intraarticular fracture through the left C6-C7 facet joint. The fractures were classified as unstable cervical spine fractures equivalent to an Abbreviated Injury Scale (AIS) grade 3 injury [14]. The patient received treatment at the hospital consisting of conservative treatment with a cervical spine collar for six weeks. Seven weeks after the first CT scan a repeated CT scan revealed worsened subluxation of the facet joints bilaterally with increasing kyphotic angulation and increased distance between the spinous processes. A third CT scan nine days later described a localized sharp kyphosis at C6-7 with significant angulation and a minor anterior spondylolisthesis. There was healing ossification of the subluxated fracture on the right side at C6-7. Due to the findings on the CT scans the patient was offered spinal surgery involving fixation of the affected area in order to ensure healing. Stabilizing osteosynthesis of the cervical spine was successfully performed approximately 3 months after the initial traffic crash. Following the surgery the patient improved significantly over the following months. Control x-rays were performed 3 months after surgery revealing persistent kyphosis but adequate healing of the fractures and surgical sites (Fig. 3). The patient suffered sequelae consisting of reduced cervical spine mobility and stiffness, and frequent neck pain with a graded disability of 12.5 %. The court sentenced the driver of the vehicle a fine and conditional disqualification from driving. No further legal actions were taken.

This case report concerns a case from a chiropractic practice where a bicyclist presented with cervical spine fractures following a bicycle crash that had been undiagnosed for 18 days despite several medical and chiropractic consultations. Following diagnostic imaging evaluation she underwent stabilizing surgery with a moderate outcome.

Bicyclists are vulnerable road users and are likely to get injured when involved in crashes [7]. When this happens the casualty most often suffers short-lived symptoms from minor contusions, lacerations and abrasions [15]. In moderate severity injuries dislocations and distortions are seen, whereas severe injuries include fractures, spinal cord injuries, intracranial injuries and injuries to the thorax and abdomen [1, 5–7, 15]. The most common injury locations are the extremities, and when controlling for injury severity the head injuries are the most common potentially fatal (AIS4+) injuries [6, 15]. Cervical spine fractures are rare following bicycle crashes affecting less than 1 % of all bicycle crash victims [1, 6, 15], with a significantly increased prevalence when a head or brain injury is present [6]. A recent meta-analysis found helmet use to increase the risk of cervical spine injury alone [3]. However, there are some conflicting reports concerning the evidence of helmet use and the risk of cervical spine injury [1, 6, 15]. In the reported case the casualty, who wore a bicycle helmet, suffered serious cervical spine injuries but no head injuries. Hence, she clearly benefitted from the proven protective effects of the helmet as this was broken due to the impact sustained during the crash [1–5]. The cervical spine fractures were most likely the consequences of the high-energy impact transferred from the initial point of contact via the helmet to the cervical spine. The important role on safety and injury prevention of bicycle helmets is clear [1–5]. Consequently, many countries have implemented this knowledge into national laws and recommendations. However, mandatory use of helmets is only enacted in a few European countries and affects only children and young people [13].

**Fig. 4:**
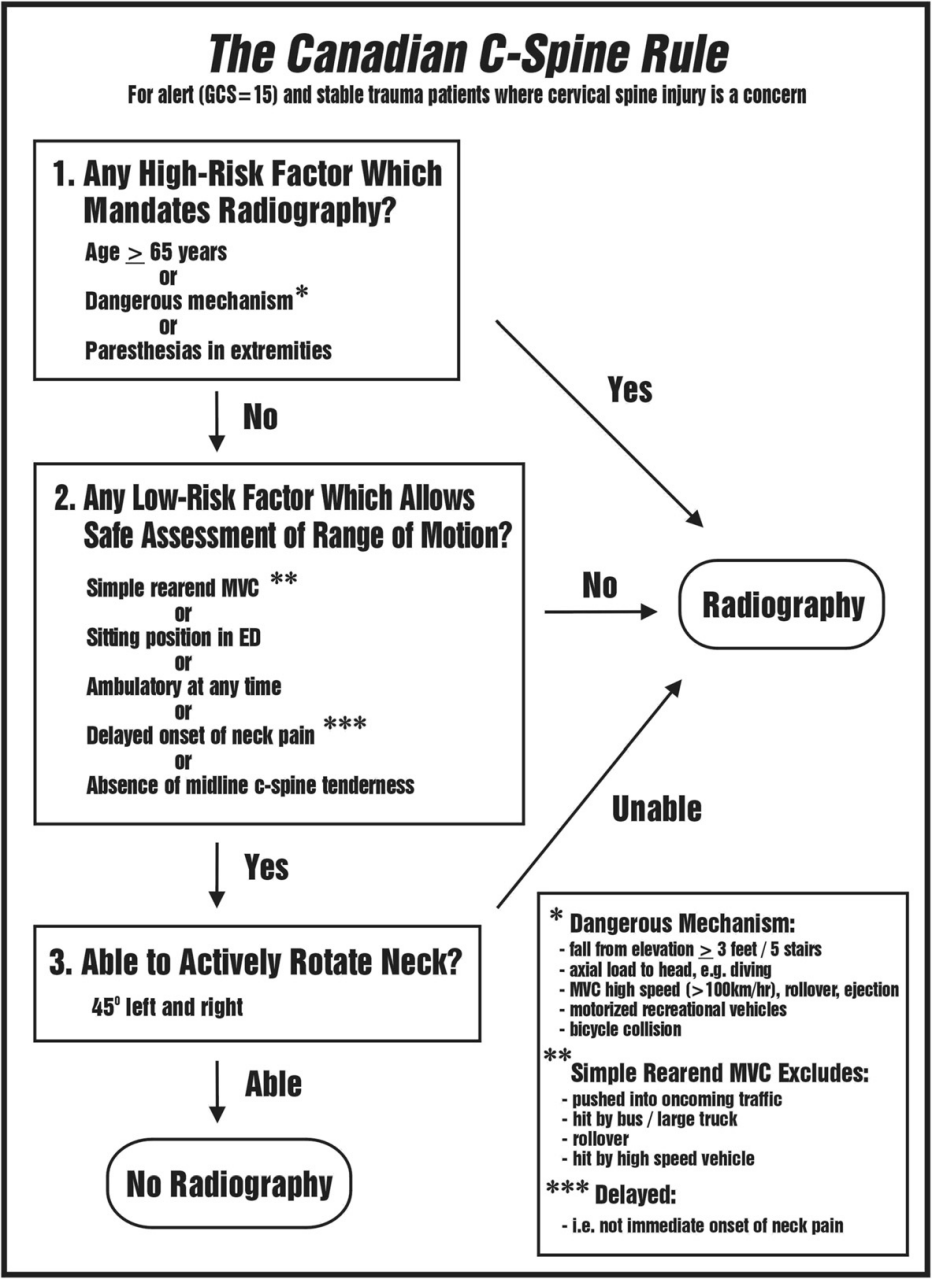
The Canadian C-Spine Rule. Reuse with permission from the author [16]

As this case report illustrates, an ED evaluation does not necessarily guarantee sufficient diagnostic evaluation. Hence, chiropractors must be able to conduct such evaluation competently irrespective of any prior examination. This is particularly relevant when managing patients involved in high-energy trauma where the risk of serious spinal injury is high. An extremely important part of such evaluation is the immediate and unrestricted access to radiological examination. In Denmark it is mandatory for chiropractors to have access to radiological equipment, although not all practices have in-house facilities. According to clinical decision strategies, x-rays should be obtained in all cases where there is suspicion of cervical spine injury, in particular when the conscious patient has been involved in a high-energy trauma similar to the reported case. Several guidelines are widely available including in particular the Canadian C-Spine rule (CCR) [16] and the National Emergency X-Radiography Utilization Study low-risk criteria (NEXUS) [17], and the protocols are endorsed by the Royal College of Radiologists [18] and the American College of Radiology [19]. Furthermore, similar evidence based practice guidelines have been provided for chiropractors [20, 21]. In clinical settings the CCR (Fig. 4) have been shown to be superior to the NEXUS criteria with regard to diagnostic accuracy [22, 23]. Hence, using such available guidelines the risk of missing cervical spine fractures is reduced significantly. Why several clinicians chose not to request a diagnostic imaging evaluation of the casualty presented despite her complaints of cervical spine stiffness and pain following the high-energy crash is not known. Apparently, neither the history nor the clinical examinations raised the suspicion of serious cervical spine injury at the ED, GP or the first chiropractor. Hence, it can be speculated whether the imaging guidelines had been utilized during any of these consultations.

When the spinal fractures were detected the patient had to be referred for hospital care. At the time of the reported case, no formal collaboration existed between the chiropractors in private practice and the hospitals with regard to referral of acute spinal injury. Therefore, the patient had to be referred by the GP. However, more recently, a direct referral option has been established in some regions of the country, e.g. Central Region Denmark, which enables the chiropractors to refer directly to the ED via the visitation procedures present at the local hospital. Treatment of acute cervical spine fractures follows relevant guidelines and do not involve the chiropractor except for the potential diagnostic role as in the presented case. However, following healing of fractures chiropractic treatment may become relevant in order to restore biomechanical function, improve physical loading capability and reduce pain. Hence, a previous serious spinal injury does not contraindicate future chiropractic care. On the contrary, chiropractors can contribute to the management and rehabilitation of patients with spinal injury following many types of crashes and accident, including bicycle crashes.

## Conclusions

The presented case is an example of the serious spinal injuries bicyclists may suffer when involved in high-energy traffic crashes despite wearing a bicycle helmet. The casualty experienced delayed diagnosis of the injuries despite several medical and chiropractic consultations. Hence, this paper highlights the necessity for clinicians, including chiropractors, to be familiar with clinical guidelines involving diagnostic imaging, when managing trauma patients. In this context, it is imperative that chiropractors have immediate and direct access to radiological procedures with no unnecessary delay. Furthermore, clearly defined and easy accessible referral schemes from primary chiropractic care settings to emergency departments must be available. Chiropractors are clinically competent to examine and diagnose, including radiologically evaluate, patients who have been injured in traffic crashes. This is a prerequisite for optimal management and rehabilitation of spinal injured patients following many types of crashes and accident, including bicycle crashes.

## Abbreviations

APLC: Anterior-posterior lower cervical
APOM: Anterior-posterior open mouth
CCR: Canadian C-Spine rule
CT: Computed Tomography
ED: Emergency departments
GP: General Practitioner
NEXUS: National Emergency X-Radiography Utilization Study

## References

[1] Amoros E, Chiron M, Martin JL, Thelot B, Laumon B (2012). Bicycle helmet wearing and the risk of head, face, and neck injury: a French case--control study based on a road trauma registry. Inj Prev.

[2] Attewell RG, Glase K, McFadden M (2001). Bicycle helmet efficacy: a meta-analysis. Accid Anal Prev.

[3] Elvik R (2013). Corrigendum to: “Publication bias and time-trend bias in meta-analysis of bicycle helmet efficacy: a re-analysis of Attewell, Glase and McFadden, 2001” [Accid. Anal. Prev. 43 (2011) 1245–1251]. Accid Anal Prev.

[4] Bambach MR, Mitchell RJ, Grzebieta RH, Olivier J (2013). The effectiveness of helmets in bicycle collisions with motor vehicles: a case-control study. Accid Anal Prev.

[5] Persaud N, Coleman E, Zwolakowski D, Lauwers B, Cass D (2012). Nonuse of bicycle helmets and risk of fatal head injury: a proportional mortality, case-control study. CMAJ.

[6] Rivara FP, Thompson DC, Thompson RS (1997). Epidemiology of bicycle injuries and risk factors for serious injury. Inj Prev.

[7] Møller H, Damm M, Laursen B (2012). Ulykker i Danmark 1990–2009.

[8] Danmarks Statistik (1998). Road traffic accidents (*Færdselsuheld*).

[9] World Health Organization (2015). Global status report on road safety.

[10] Forsse A, Eskesen V, Springborg JB (2015). Bicycle helmet prevents brain damage. Ugeskr Laeger.

[11] Danmarks Statistik (2015). UHELDK1: Tilskadekomne og dræbte i færdselsuheld efter område, personskade, indblandede transportmidler, alder og køn.

[12] Rådet for Sikker Trafik (2013). Hver ulykke er én for meget- et fælles ansvar. Færdselssikkerhedskommisionens nationale handlingsplan 2013–2020.

[13] European Transport Safety Council (2015). Making walking and cycling on Europe’s roads safer.

[14] Gennarelli TA, Wodzin E (2008). The Abbreviated Injury Scale 2005. Update 2008.

[15] Amoros E, Chiron M, Thelot B, Laumon B (2011). The injury epidemiology of cyclists based on a road trauma registry. BMC Public Health.

[16] Stiell IG, Wells GA, Vandemheen KL, Clement CM, Lesiuk H, De Maio VJ (2001). The Canadian C-spine rule for radiography in alert and stable trauma patients. JAMA.

[17] Hoffman JR, Mower WR, Wolfson AB, Todd KH, Zucker MI (2000). Validity of a set of clinical criteria to rule out injury to the cervical spine in patients with blunt trauma. National Emergency X-Radiography Utilization Study Group. N Engl J Med.

[18] The Royal College of Radiologists (2012). iRefer: making the best use of clinical radiology.

[19] American College of Radiology. American College of Radiology ACR Appropriateness Criteria. American College of Radiology. 2012. https://acsearch.acr.org/docs/69359/Narrative/. Accessed 9 Aug 2016.

[20] Bussieres AE, Taylor JA, Peterson C (2008). Diagnostic imaging practice guidelines for musculoskeletal complaints in adults-an evidence-based approach-part 3: spinal disorders. J Manipulative Physiol Ther.

[21] Jensen TS, Torfing T, Gregersen HE, Lundorf E, Morsel L, Vendrup SS (2014). Kliniske retningslinjer - Billeddiagnostiske undersøgelser af bevægeapparatet.

[22] Michaleff ZA, Maher CG, Verhagen AP, Rebbeck T, Lin CW (2012). Accuracy of the Canadian C-spine rule and NEXUS to screen for clinically important cervical spine injury in patients following blunt trauma: a systematic review. CMAJ.

[23] Stiell IG, Clement CM, McKnight RD, Brison R, Schull MJ, Rowe BH (2003). The Canadian C-spine rule versus the NEXUS low-risk criteria in patients with trauma. N Engl J Med.

